# Rational choice and political imaging in mining areas: a case study of legislative elections in East Luwu

**DOI:** 10.3389/fsoc.2025.1564925

**Published:** 2025-04-25

**Authors:** Rahmat Muhammad, Ridwan Syam, Irfan Yahya, Patta Hindi Asis

**Affiliations:** ^1^Department of Sociology, Hasanuddin University, Makassar, Indonesia; ^2^Faculty of Social and Political Sciences, Universitas Muhammadiyah Kendari, Kendari, Southeast Sulawesi, Indonesia

**Keywords:** rational choice, voter behavior, political imaging, mining areas, political sociology

## Abstract

The 2024 legislative elections in Indonesia present an important opportunity to examine the dynamics of voters’ rational choices in the context of developing democracy. This study investigates differences in voters’ rational choices regarding the image-building actions of legislative candidates in the East Luwu mining industry, focusing on the differences between inner and outer mining circles. Using a qualitative approach with in-depth interviews of ten key informants, this research reveals significant variations in voter rationality and their responses to candidates’ image-building strategies. The findings indicate that voters within the inner mining circle exhibit greater rationality and critical thinking in their decision-making. They tend to evaluate candidates based on their track records, policy proposals, and overall competence—reflecting a more educated, policy-focused, and socially conscious electorate with high social capital. Conversely, in the outer mining circle, voters are influenced more by primordial factors and short-term incentives, so this is driven by kinship, short-term incentives, low info access, and traditional loyalties. In terms of differences in education, access to information, campaign methods, and voter priorities as indicators of voters. Nevertheless, the roles of social capital and social networks remained significant in both areas. These differences reflect variations in education level, welfare, and access to information. This study contributes to the development of rational choice theory by demonstrating how structural factors can influence political rationality at the local level. Important implications include the need for different campaign strategies and political education efforts that consider specific characteristics of local communities.

## Introduction

The 2024 legislative elections in Indonesia present a unique opportunity to examine the dynamics of voter rationality in the context of a developing democracy. As a country with high ethnic, cultural, and socioeconomic diversity, Indonesia presents a complex and challenging political landscape to understand through the lens of conventional rational choice theory. Although this theory has long been a foundation for understanding voter behavior, recent scholarly debates have questioned its application in complex political landscapes, particularly in developing countries (Blais and Daoust, 2020). The assumption that voters are purely self-interested actors who maximize personal utility has been challenged by empirical evidence showing a more nuanced decision-making process influenced by social, cultural, and emotional factors (Achen and Bartels, 2017).

This theoretical uncertainty calls for a re-examination of rational choices in the context of Indonesia’s diverse electorate, where traditional patronage networks coexist with modernizing political institutions (Aspinall and Berenschot, 2019). This dynamic becomes increasingly complex with variations in development levels and socioeconomic characteristics across regions in Indonesia, potentially affecting voter rationality in different ways. This situation creates challenges and opportunities to enrich our understanding of voter behavior in developing democracies.

Empirically, there is a growing gap between the ideal notion of informed, policy-oriented voters and the reality of voter behavior in many parts of Indonesia. Although democratic reforms have aimed to foster a more engaged and critical electorate, studies show that many voters continue to base their choices on short-term material incentives or personal loyalties, rather than long-term policy considerations (Muhtadi, 2019). This phenomenon not only reflects the limitations of rational choice theory in explaining voter behavior in Indonesia but also indicates the presence of contextual factors that need to be considered when analyzing voter behavior.

The gap between the aspirational goals of democratic consolidation and the pragmatic realities of voter decision making poses significant challenges for Indonesia’s political development (Fossati, 2019). On one hand, efforts to improve the quality of democracy demand more rational and policy-oriented voters. On the other hand, existing socio-economic realities and political culture often push voters to act based on short-term considerations or primordial loyalties. Understanding this dynamic is crucial for designing effective strategies to enhance citizens’ political participation.

Moreover, the increasing role of social media and digital campaigning introduces new variables into the rational choice equation, potentially altering how voters access and process information regarding legislative candidates (Tapsell, 2021). The digital era has opened wider access to political information, but at the same time, it has also created new challenges in the form of spreading inaccurate or manipulative information. This phenomenon adds complexity to understanding the voter decision-making process and demands a more nuanced approach for analyzing voter behavior in the digital age.

In this context, this research fills a gap in the study of voter behavior in Indonesia by focusing specifically on the differences in rational choices between voters in inner and outer mining circles. The choice to focus on mining areas is based on the consideration that these regions often have unique socioeconomic characteristics, with significant welfare disparities between areas close to mining activities (inner circle) and more distant areas (outer circle). This condition provides a natural laboratory to test how differences in socioeconomic conditions within one industrial area can affect voter rationality.

Previous studies have extensively discussed voter behavior in national (Aspinall et al., 2018; Fossati, 2019) and urban contexts (Warburton, 2020), but none have specifically examined the dynamics of rational choice in extractive industry areas. Past research has tended to focus on money politics (Aspinall and Berenschot, 2019; Muhtadi, 2019) or the influence of social media (Lim, 2017; Tapsell, 2021), but has not explored how differences in socioeconomic conditions within one industrial area can affect voter rationality. Thus, this research not only fills a gap in the literature, but also provides new insights into how the local context can influence the application of rational choice theory in voter behavior.

This study also expands our understanding of rational choice theory by incorporating sociological perspectives, addressing the gap identified by Grzymala-Busse (2019) and Becker (2023) regarding the need for a more integrated approach to understanding voter behavior in developing democracies. From a rational choice perspective, the preference for short-term material incentives over long-term policy considerations suggests that voters may not always act in their best long-term interests. This behavior could be attributed to factors such as limited access to information, high levels of economic insecurity, or a lack of trust in the political system’s ability to deliver long-term benefits (Mares and Young, 2019). This approach allows for a more comprehensive analysis of how structural factors, such as the level of economic development and access to education, interact with social norms and culture to shape voters’ preferences and decisions.

Unlike previous studies that generally used quantitative approaches (Dunning et al., 2019; Suggs, 2021) or macro analysis (Mietzner, 2020; Power, 2018), this study adopted a qualitative approach with in-depth interviews. This methodological choice is based on the need to understand the voter decision-making process in the specific context of mining areas. The qualitative approach allows for a more nuanced exploration of voter motivations and considerations, something not fully revealed in survey-based studies, such as those conducted by Moslehpour et al. (2024) and Hedman and Ufen (2010).

Furthermore, the use of James S. Coleman’s rational choice theory combined with contextual analysis of mining areas provides a new perspective that has not been explored in the previous literature on voter behavior in Indonesia. This approach allows researchers to explore not only how voters make decisions but also how socio-economic and cultural contexts influence this decision-making process.

Thus, this research aims to deeply understand the voter decision-making process in the specific context of East Luwu’s mining area, focusing on the differences between the inner and outer mining circles. Through a qualitative approach with in-depth interviews, this research seeks to uncover the nuances and complexities of voters’ rational choices that might have been overlooked in previous survey-based studies. The results of this research are expected to provide a more comprehensive understanding of the dynamics of voters’ rational choices in mining industry areas, as well as their implications for political campaign strategies and voter education efforts in these regions.

Moreover, the findings of this study are expected to contribute to the development of theory and practice in the field of election and democracy studies in developing countries. By revealing the nuances and complexities of voters’ rational choices in mining areas, this study can help identify key factors that need to be considered when designing policies and programs aimed at improving the quality of citizens’ political participation. Ultimately, a better understanding of these dynamics can assist in formulating effective strategies to strengthen democracy in Indonesia and other developing countries.

## Methods

This study was conducted in East Luwu Regency, South Sulawesi Province, Indonesia, an area known for its significant nickel mining industry. This regency was chosen as the research location because of its unique characteristics as a mining area, creating an ideal context for investigating differences in voters’ rational choices. According to the East Luwu Statistics Bureau (BPS) data from 2021, the regency covers an area of 6,944.88 km^2^ with a population of 300,770, and exhibits varied socio-economic landscapes between areas close to mining operations (inner mining circle) and those further away (outer mining circle).

For the purposes of this study, Nuha District was selected to represent the inner mining circle, whereas Malili District represented the outer mining circle. This selection was based on differences in socioeconomic characteristics between the two areas. Nuha District has higher levels of education and welfare, with a literacy rate of 99.54% and a Human Development Index (HDI) of 76.32. In contrast, as data from the BPS indicate, Malili District has relatively lower indicators, with a literacy rate of 97.89% and an HDI of 72.45. These differences allow researchers to analyze how variations in socioeconomic conditions within a single regency can influence patterns of voters’ rational choices in the context of legislative elections.

This study adopts a qualitative approach with a case study design to gain an in-depth understanding of the phenomenon of voters’ rational choice in the context of legislative elections in mining areas. The qualitative approach was chosen for its ability to uncover nuances and complexities in voter decision making that are difficult to capture through quantitative methods (Creswell and Poth, 2016). This case study focuses on the specific context of the East Luwu mining area, allowing for an in-depth exploration of the unique social, economic, and political dynamics in this region (Yin, 2017). The selection of this method aligns with recent trends in voter behavior research, which emphasize the importance of understanding local contexts in shaping political preferences (Fossati et al., 2020).

Data collection was conducted through semi-structured interviews with 10 key informants, consisting of five informants from the inner mining circle (Nuha District) and five informants from the outer mining circle (Malili District). The informants were selected using a purposive sampling technique to ensure representation of various perspectives and backgrounds (Patton, 2014). Interviews were conducted face-to-face, lasting approximately 60 min per participant. The profile of each informant can be seen in Table 1. This approach allows researchers to deeply explore voters’ understanding, motivations, and considerations in their socio-political context, in line with recommendations (Aspinall and Berenschot, 2019) for research on voter behavior in Indonesia.

**Table 1:** Profile of research informant.

Informant location	Initial name	Age	Gender	Work
Malili sub-district (outer ring of mine)	SJ	60	Male	Self-employed
	SA	62	Male	Contractor Entrepreneur
	KS	51	Male	Civil servants
	IA	48	Female	Self-employed
	IJ	55	Male	Merchant
Nuha sub-district (inner ring of mine)	IF	52	Male	Civil servants
	NM	45	Female	Private Employee
	JH	50	Female	Civil servants
	CT	60	Male	Private Employee
	BE	48	Male	Contractor Entrepreneur

Interview data collection was conducted with the full consent of the informants. Prior to the interviews, informants were provided with an explanation regarding the ethical approval letter. This research obtained ethical approval from the Research Ethics Committee (KeP) of Hasanuddin University, number 816/UN4.6.4.5.31/PP36/2024, which confirmed that the research proposal and protocol had undergone ethical review and were approved. During this process, the researcher explained to the informants the importance of informed consent. Interestingly, the majority of informants were more comfortable providing verbal rather than written consent. They viewed themselves as community representatives and considered this status as sufficient indication of their competence without the need for written documentation. This preference reflects the tendency in Indonesian culture to value verbal agreements and avoid excessive formality in communication. Informants generally felt that verbal consent was more aligned with their social practices and adequate as a form of permission. This approach was deemed more efficient and in harmony with societal norms that emphasize trust and verbal agreements over written documentation. In the context of this research, verbal consent was treated as equivalent to written consent, respecting the cultural preferences of the informants while maintaining the integrity of the research process.

Data analysis employed a thematic analysis method, which involved a systematic coding process to identify patterns and main themes in the interview data (Braun and Clarke, 2021). This process included interview transcription, initial coding, theme development, and interpretation of the results. To enhance the validity and reliability of the research, data triangulation was conducted by comparing the interview results with the relevant documentation and field observations (Noble and Heale, 2019). This analytical approach allows researchers to uncover the nuances of voters’ rational choices that might be missed in quantitative analysis, in line with Muhtadi’s (2019) the argument about the importance of contextual understanding in studying voter behavior in Indonesia.

## Results and discussion

### Image-building actions of legislative candidates in mining areas

Legislative candidates use various methods to attract voter sympathy in the mining areas. Based on the field findings, there are differences in the image-building strategies employed by candidates in the inner and outer mining circles. These differences reflect variations in socioeconomic conditions and voter characteristics in both areas.

In the inner mining circle, particularly in Nuha District, image building of legislative candidates is more often done through group communication channels and direct interaction with the community. As confirmed by one informant:

“From what I see, firstly, what is often done are steps of building relationships in various moments, such as at parties, religious gatherings. Second, billboards are placed. However, relationship-building is the most important, because, on average, candidates always take time to attend crowded events such as wedding parties, condolence visits, and religious study groups. So, they prioritize direct interaction.”

This approach reflects the characteristics of the community in the inner mining circle, which tends to be more critical and rational in assessing legislative candidates. They not only look at the candidate’s popularity through print media, but also assess the candidate’s closeness and direct interaction with the community. This aligns with the findings by Aspinall and Berenschot (2019) showing that in areas with higher levels of education and welfare, voters tend to be more critical in assessing candidates.

On the other hand, in the outer mining circle, such as in the Malili District, image building of legislative candidates is still dominated by the use of print media, especially billboards. As expressed by one informant:

“Still billboards, because here people are less affluent and, less knowledgeable about using digital media. Therefore, most of their image building is done through billboards only. Generally, through billboards.”

The use of billboards as the primary image-building method in the outer mining circle indicates a digital divide and differences in education levels between the two areas. This is in line with Tapsell (2021) research showing that, in areas with limited digital access, conventional campaign methods are still dominant.

Interestingly, although social media is becoming increasingly popular, its effectiveness in influencing voter choice remains limited, especially in the outer mining circle. As expressed by the informant:

“In my opinion, the tools used by legislative candidates are popularity, not electability. It’s only about popularity, just wanting to convey that I am here, I am present, I am running, but not for their electability.”

This finding shows that, while social media can increase candidate visibility, it does not necessarily translate directly into voter support. This aligns with the finding (Koc-Michalska et al., 2016) that the effectiveness of digital campaigns highly depends on the socioeconomic context and local political culture.

Besides image-building methods, the issues raised by legislative candidates also vary between inner and outer mining circles. In the inner mining circle, candidates tend to focus on substantive issues and work programs. As expressed by the informant:

“What the community favors most are candidates who do not just make promises but provide evidence. This is what the community really needs, especially in Nuha, based on what I observe. People here are avid readers, and their education level is also quite high.”

However, in the outer mining circle, primordial issues such as ethnicity and kinship still play an important role. As stated by the informant:

“There are two factors. The first is an individual’s intellect and intelligence. That person is able to, well, and attract support. The second factor I see is their culture, their lineage, if within that scope they have many family members.”

These differences reflect variations in the social structure and level of modernization between the two areas. This finding aligns with the study by Aspinall et al. (2017), which shows that in more economically advanced areas, voters tend to be more program-oriented, whereas in less developed areas, primordial factors remain dominant.

From the perspective of rational choice theory, the differences in image-building strategies and voter responses in both areas can be explained by different rational calculations. In the inner mining circle, where education and welfare levels are relatively high, voters tend to maximize their utility by choosing candidates deemed most competent and having relevant programs. This encourages legislative candidates to build their image through direct interaction and an emphasis on work programs.

Conversely, in the outer mining circle, where socioeconomic conditions are relatively less developed, voters tend to maximize their short-term utility. This is reflected by the strong influence of primordial factors and political transactions. As expressed by the informant:

“Excuse me, regarding the issue of vision and mission, in my view, if we say 100 percent, only about 10 percent talk about vision and mission. Eh, why? Because that vision and mission will disappear on its own later, and people do not talk about the vision and mission on D-1, they do not discuss that. There are two factors that I see here, excuse me, two things that emerge: how much (money) and who they are, meaning their family lineage.”

This finding aligns with the argument (Gans-Morse et al., 2014) that, in areas with low levels of welfare, voters tend to be more responsive to short-term incentives in elections.

Interestingly, although there are differences in image-building strategies, in both the inner and outer mining circles, the roles of social capital and social networks remain significant. This is reflected in the importance of relationship building and the presence of legislative candidates at various social events. This phenomenon can be explained through the concept of “embedded rationality” proposed by Granovetter (1985), where an individual’s rational choice cannot be separated from their social context.

The image-building actions of legislative candidates in mining areas show variations that reflect differences in socioeconomic characteristics between inner and outer mining circles. In the inner circle, image-building is more oriented toward substance and direct interaction, whereas in the outer circle, conventional methods and primordial factors are still dominant. These findings affirm the importance of understanding the local context in analyzing political campaign strategies and voter behavior, while also showing that rational choice theory needs to be interpreted by considering variations in socioeconomic conditions and local political culture.

### Voter rationality in legislative candidate image

Rational choice theory emphasizes that the actions taken by individuals have goals and are determined by values or preferences (choices). In legislative elections, voter rationality is reflected in how voters assess and choose candidates based on certain considerations. This research reveals variations in voter rationality between the inner and outer mining circle areas in the East Luwu Regency, reflected in the differences in their preferences and considerations in assessing the image of legislative candidates.

In the inner mining circle, particularly in Nuha District, voters tend to be more rational and critical of assessing candidates. They give more consideration to the track record, programs, and candidates’ ability to represent community aspirations. As expressed by one informant:

“In Nuha, from what I’ve heard, though I have not been here long. Regardless, what is most favored by the community are candidates who do not just make promises but also provide evidence. This is what is really needed by the community, especially in Nuha, which is what I see. The people here are avid readers, and their education levels are also quite high.”

This finding aligns with the argument (Aspinall and Berenschot, 2019) that voters tend to be more critical and program oriented in areas with higher levels of education and welfare. This also reflects the concept of “sophisticated voting” proposed by Dassonneville and Dejaeghere (2014), where more educated voters tend to vote based on a more complex evaluation of candidates.

Furthermore, voters in the inner mining circle appear more resistant to money politics and empty promises. One informant stated:

“That has little influence, no, not here, not here.”

When asked about the influence of “envelope content” or financial incentives on voting. This indicates that voters in this area tend to prioritize rational considerations over short-term gains. This finding supports the argument that the effectiveness of Kitschelt (2007) political patronage tends to decrease in areas with higher levels of economic development.

On the other hand, in the outer mining circle, such as in the Malili District, voter rationality appears to be influenced more by primordial factors and short-term considerations. As expressed by the informant:

“Excuse me, I see the issue of vision and mission, in my view, if we say 100 percent, only about 10 percent talk about vision and mission. Eh, because why? That vision and mission will disappear by itself later, and people do not talk about vision and mission on D-1; they do not talk about that. There are two factors that I see here, excuse me, there are two things that emerge, how much (money) and who they are, meaning their family lineage.”

This finding indicates that, in areas with lower levels of welfare, voters tend to be more responsive to short-term incentives and primordial ties. This aligns with the argument of Gans-Morse et al. (2014) that patronage and clientelist practices tend to be more effective in areas with higher levels of poverty.

Interestingly, despite differences in levels of rationality, both in inner and outer mining circles, the role of social capital and social networks remains significant in influencing voter choice. As expressed by the informant:

“The top ranking is in social relationships, social relations, yes, that cannot be beaten. And family too, lineage, right, so besides that, if you advance your lineage, whoever has a large family lineage and good social investment in that family, that’s it.”

This phenomenon can be explained through the concept of “embedded rationality” proposed by Granovetter (1985), where an individual’s rational choice cannot be separated from their social context. In the context of East Luwu, social networks and kinship still play important roles in shaping voter preferences, even among more rational voters.

The difference in voter rationality between the inner and outer mining circles is also reflected in how they respond to the candidate image-building strategies. In the inner mining circle, voters tend to be more critical of superficial image-building. One informant stated:

“Academic titles count, but lineage does not.”

This shows that voters in this area value achievement and competence more than ascriptive factors, such as lineage. This finding aligns with Dalton’s (2018) the argument that modernization and increased education tend to shift voters’ preferences from traditional loyalties to more rational evaluations of candidate performance and competence.

Conversely, primordial and transactional image building is still quite effective in the outer mining circle. As expressed by the informant:

“There are two things, the first is the person’s intellect, their intelligence. That person is able to, um, and attract support. Second, I also look at their culture, and lineage; within that scope, they have many family members. Besides that, intelligence, they also have many family members, their culture.”

This finding underscores the importance of understanding the sociocultural context in analyzing voter behavior. As argued by Schaffer (2007), patronage and clientelism practices are not just economic transactions, but are also embedded in local social and cultural norms.

The difference in voter rationality is also reflected in how voters respond to the use of social media and digital technology in campaigns. Although the use of social media in the inner mining circle is increasing, its effectiveness in influencing voter choices is still limited. One informant stated:

“Yes, because they like media, so those who are diligent can see the posts that are there, it’s up to them to choose which ones they like.”

This indicates that voters in this area tend to be more selective about filtering information from social media. This finding aligns with a study showing (Enli and Rosenberg, 2018) that, in countries with high levels of digital literacy, voters tend to be more critical of political information on social media.

However, in the outer mining circle, the use of social media in political campaigns does not seem to be effective. As expressed by the informant:

“The fact is, in the field, we see in the villages, even nearby here no one opens it.”

This indicates that a digital divide affects the effectiveness of online campaigns in this area. This finding supports (Strömberg, 2015) the argument that gaps in access to and use of technology can reinforce the existing political inequalities.

From the perspective of rational choice theory, the differences in voter rationality between inner and outer mining circles can be understood as a result of differences in incentive structures and constraints faced by voters in both areas. In the inner mining circle, with higher levels of education and welfare, voters have better access to information and resources that allow them to make more complex evaluations of the candidates. This aligns with Bali et al. (2020) the argument that rational voters try to maximize their utility by choosing candidates deemed most capable of meeting their interests.

Conversely, in the outer mining circle, where welfare levels are lower and access to information is limited, voters tend to rely more on social networks and short-term incentives to make decisions. This can be understood as a rational strategy to maximize immediate benefits in the context of limited resources and information. As argued by Kitschelt (2007), under conditions of economic uncertainty, voters may consider it more rational to accept immediate benefits rather than wait for uncertain long-term political promises.

However, it is important to note that although there are differences in the levels of rationality, voters in both the inner and outer mining circles are not entirely “rational” in the classical sense of rational choice theory. Factors such as social ties, cultural identity, and local norms continue to play an important role in shaping voter preferences. This shows that voter rationality needs to be understood in a broader sociocultural context, in line with the concept of “bounded rationality” proposed by Simon (1997).

Voter rationality in assessing the image of legislative candidates in the East Luwu Regency shows significant variation between inner and outer mining circles. Voters in the inner mining circle tend to be more rational and critical, with preferences oriented toward candidate programs and competencies. Conversely, voters in the outer mining circle are influenced more by primordial factors and short-term considerations. However, in both areas, the role of social capital and social networks remains significant in shaping voters’ preferences.

These findings have important implications for candidate legislative campaign strategies and political education efforts in the region. In the inner mining circle, candidates need to focus on their programs and track records, whereas in the outer mining circle, a more personal and community-based approach might be more effective. More broadly, these findings also underscore the importance of understanding local variations in voter behavior and political rationality, as well as how structural factors, such as levels of economic development and access to education, can influence electoral political dynamics.

## Conclusion

This study reveals significant differences in voters’ rational choices and their responses to legislative candidates’ image-building strategies between the inner and outer mining circle areas in the East Luwu Regency. In the inner mining circle, voters tend to be more rational and critical, giving more consideration to candidates’ track records, programs, and ability to represent community aspirations. They are also more resistant to money politics and superficial image-building. Conversely, in the outer mining circle, voters are influenced more by primordial factors, short-term considerations, and material incentives. These differences reflect variations in education levels, welfare, and access to information between the two areas.

These findings have important implications for our understanding of electoral political dynamics in mining industry areas and highlight the importance of considering local socioeconomic contexts in analyzing voter behavior. Although there are differences in levels of rationality, the role of social capital and social networks remains significant in both areas, indicating that voters’ rational choices need to be understood within a broader sociocultural context. This research also underscores the need for different campaign strategies for each area, as well as the importance of political education efforts that consider the specific characteristics of local communities. More broadly, this study contributes to the development of rational choice theory by demonstrating how structural factors such as levels of economic development and access to education can influence political rationality at the local level.

However, it is important to acknowledge the limitations of this study, particularly in terms of its longitudinal scope. As this research provides a snapshot of voter behavior during a specific election cycle, future studies could benefit from adopting a longitudinal approach to examine how voter preferences and decision-making processes evolve over time in response to changing socioeconomic conditions in mining areas. Such an approach would offer a more comprehensive understanding of the dynamics of voter rationality and the factors that shape it in the long run.

## Data Availability

The datasets presented in this study can be found in online repositories. The names of the repository/repositories and accession number(s) can be found in the article/supplementary material.
